# Delayed postoperative spinal epidural hematoma after anterior cervical discectomy and fusion: A case report

**DOI:** 10.3389/fsurg.2022.1005462

**Published:** 2022-09-26

**Authors:** Wenbin Xu, Jiandong Guo, Jinjin Zhu, Xing Zhao, Iranmanesh Yasaman, Jian Chen, Jiying Wang, Shunwu Fan, Xiangqian Fang

**Affiliations:** ^1^ Department of Orthopedic Surgery, Sir Run Run Shaw Hospital, Zhejiang University School of Medicine, Hangzhou, China; ^2^ Key Laboratory of Musculoskeletal System Degeneration and Regeneration Translational Research of Zhejiang, Hangzhou, China; ^3^ Department of Orthopaedics, Hangzhou Ninth People's Hospital, Hangzhou, China; ^4^ Zhejiang University, School of Medicine, Hangzhou, China

**Keywords:** anterior cervical discectomy and fusion, spinal epidural hematoma, surgery, spine, anterior cervical discectomy

## Abstract

**Background:**

Postoperative spinal epidural hematoma (POSEH) causes rapid neurological deficits within 24 h following the operation and can be fatal. However, some POSEH symptoms manifest three days after the operation, also known as delayed POSEH (DPOSEH). Little attention has been provided upon DPOSEH owing to its rare incidence, resulting in serious consequences upon occurrence. To date, no cases of delayed POSEH after anterior cervical surgery have been reported.

**Case presentation:**

We describe a case of DPOSEH that presented with delayed neurological deficits on the fifth day after anterior cervical discectomy and fusion (ACDF) surgery. Methylprednisolone was administered but showed no efficacy. MR revealed low T1 and strip long T2 signals located behind discs. After emergency surgical decompression, the patient's muscle strength returned to the preoperative state. However, his muscle strength decreased again on the seventh postoperative day, and the patient's family refused further surgery. Nine months after ACDF, the patient died of septic shock and respiratory failure.

**Conclusions:**

DPOSEH can occur after three days or more following anterior cervical surgery; hence, monitoring of neurological function is suggested to be extended. Complete evaluation of risk factors, timely recognition, and differentiation of neurological symptoms are required for spine surgery. In the case of DPOSEH, methylprednisolone can be administered reasonably during the transition period. However, if there is no resolution of symptoms, emergency surgery should be performed as soon as possible.

## Introduction

Cervical spine surgery is a classic treatment for cervical degenerative disc disease, trauma, infection, and tumors. It can be performed using anterior, posterior, combined, and minimally invasive techniques. Over the past 30 years, the number of cervical spine operations has increased dramatically. From 1992 to 2005, the number of cervical spine surgeries within the scope of medical insurance increased three times in the United States (1). In 2013, the rate of cervical spine operations was estimated at 72.2 cases/100,000 people (2). After spinal surgery, 33%–100% of patients develop a small spinal epidural hematoma (SEH) in the surgical area, most of which have no apparent clinical symptoms (3, 4). SEH is commonly observed at 16–45 years of age in the cervical and thoracic spines and at 46–75 years of age in the thoracic and lumbar spines (5).

The incidence of postoperative symptomatic spinal epidural hematoma (POSEH) is 0.1%–3% (6, 7) and is characterized by progressive paralysis, dyspnea, and fecal incontinence, resulting in high disability and death rates. In 1869, Jackson first reported symptomatic cervical SEH in a 14-year-old girl without a history of trauma or surgery (8). She was diagnosed five days after the onset of symptoms; however, the patient died 30 h later. Since then, attention to symptomatic SEH has gradually increased. Most cases of SEH occur within 24 h of the operation (9). For SEH occurring immediately after the operation, doctors may generally make a quick judgment for treatment, and the prognosis is relatively ideal. However, a few POSEH symptoms develop more than three days after the operation, also known as delayed postoperative spinal epidural hematoma (DPOSEH), the incidence of which is 0.05%–0.6% (10–12). Cases of POSEH and DPOSEH were all reported. Since DPOSEH is sporadic and rare, little attention has been paid to it, resulting in serious consequences. Therefore, it is necessary to discuss DPOSEH at a deeper level.

Studies on DPOSEH are limited, among which only posterior cervical spine surgery is involved. Uribe et al. reported that DPOSEH occurred on the third and eight days in seven patients after the operation, and two of them recovered to American Spinal Cord Injury Association grade C after posterior cervical spine surgery (11). Neo et al. reported a case of DPOSEH on the ninth day after posterior cervical spine surgery (13). A small artery rupture in the neck muscle was found during the second operation, which was considered to be related to constipation and intense activities after getting out of bed. Khan et al. reported that DPOSEH occurred on the third and ninth days after posterior cervical spine surgery in two cases, which mainly manifested as neck pain and spasm, without muscle strength reduction, and both patients completely recovered after a second operation (14). Tomii et al. reported a case of DPOSEH seven days after posterior cervical spine surgery, with a history of hypertension and poor perioperative blood pressure control. The patient's neck pain was relieved, and muscle strength of the left upper extremity recovered two months after hematoma removal (15). Among a series of posterior cases reported by ANN et al., one case of DPOSEH occurred five days after posterior cervical spine surgery (16).

To the best of our knowledge, there have been no reports of DPOSEH after cervical spine surgery using the anterior approach. This study reports the clinical characteristics, diagnosis, and treatment process of a patient with DPOSEH after anterior cervical discectomy and fusion (ACDF) surgery and discusses the risk factors, manifestations, and treatment.

## Case report

**Figure 1:**
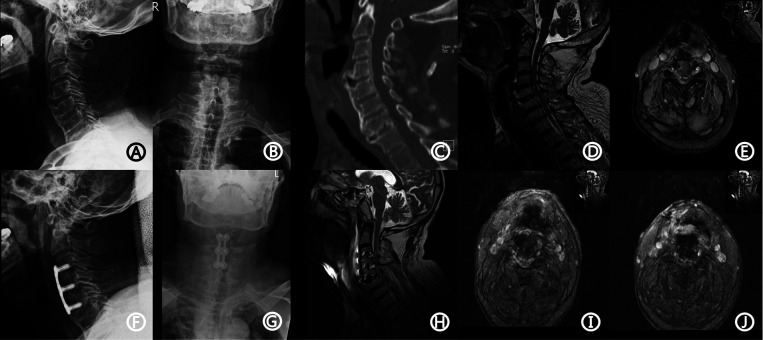
The patient's imaging data. Preoperative x-ray showed degenerative changes (**A,B**). CT showed a bone bridge in the anterior part of the vertebral body (**C**). MRI showed disc herniation at the level of C3/4 and C4/5 vertebrae, causing compression of the spinal cord and corresponding spinal canal stenosis (**D,E**). Postoperative x-ray showed the good position of the plate and interbody (**F,G**). Emergency MRI showed low T1 and strip long T2 signal located behind C3/4 and C4/5 discs, and DPOSEH was suggested (**H–J**). CT, computed tomography; MRI, magnetic resonance imaging; DPOSEH, delayed postoperative spinal epidural hematoma.

An 85-year-old man was admitted to the hospital because of numbness in both legs with an unsteady gait for two months. He had a history of hypertension for ten years with no regular treatment, gout for ten years, smoking for 30 years, and no drinking. Physical examination on admission showed a blood pressure of 121/75 mmHg and hypoesthesia of both feet, which was worse on the right side. The distal muscle strength of the right upper limb and right lower limb was grade 4. Hoffman's sign was positive on both sides. Laboratory examination showed that the RH blood group was positive, platelet count was 82 × 10^9^/L, prothrombin time was 14.4 s (range, 11.5–14.5 s), partial thromboplastin time was 29.5 s (range, 28–40 s), and international normalized ratio (INR) was 1.1 (0.9–1.1). The radiograph before the operation indicated degenerative changes in the cervical spine (Figures 1A,B). Computed tomography (CT) revealed the formation of a bone bridge in the anterior part of the vertebral body (Figure 1C).

Magnetic resonance imaging (MRI) showed disc herniation at the level of the C3/4 and C4/5 vertebrae, causing compression of the spinal cord and corresponding spinal canal stenosis (Figures 1D,E). The patient was diagnosed with cervical spondylotic myelopathy. The patient underwent anterior C3/4 and C4/5 discectomy, fusion, and internal fixation with the SYNTHES anterior cervical instrument under general anesthesia, and a drainage tube was placed before closure. The operation process was smooth with 200 ml of intraoperative hemorrhage; the postoperative radiograph is shown in Figures 1F,G. After the operation, the patient had no complaints of sensory or motor abnormalities and was treated with neck brace protection, antibiotics, and nerve nutrition. On the first day after the operation, the hemoglobin level was 11.6 g/dl (12–16 g/dl), platelet count was 75 × 10^9^/L, and INR was 1.11. On the third day after the operation, the drainage tube was removed, and the patient was permitted to walk out of bed under the protection of the neck brace.

On the fifth day after the surgery, the patient suddenly experienced limb weakness accompanied by dysuria, with grade 2 muscle strength. Emergency MRI revealed low T1 and strip long T2 signals located behind the C3/4 and C4/5 discs, and DPOSEH was suggested (Figures 1H–J). Immediately, 30 mg/kg of methylprednisolone was administered intravenously for 15 min; however, the patient's muscle strength did not improve half an hour later. Thus, emergency surgery was performed, during which the plate, screws, and the fusion device were removed. We found a blood clot in front of the dura mater in the intervertebral space, but no active bleeding was observed. The fusion device was then put back, the plate and renovation screws were installed, and two drainage tubes were placed. After the operation, the patient's muscle strength immediately recovered to grade 4, and he was transferred to the intense care unit, where methylprednisolone, mannitol, nicardipine, and other supportive treatments were administered. On the seventh day after the operation, the patient's muscle strength decreased again. The muscle strength of the left upper limb was of grade 3; right upper limb, grade 2; left lower limb, grade 2; and right lower limb, grade 1. Another immediate MRI was suggested to check for the presence of hematoma and assess the necessity of performing another surgery. However, the patient's family refused further surgeries. Another MRI in the presence of renewed motor weakness was also not done, because relatives did not want further therapy and thus, no consequence could be drawn. On the 26th day after the operation, the patient experienced dyspnea, with 88% oxygen saturation; tracheal intubation was performed. Three months after the operation, the patient underwent a tracheotomy and was placed on a ventilator. Six months after the operation, the muscle strength did not improve. The patient passed away nine months after the first operation owing to septic shock and respiratory failure.

## Discussion

### Risk factors

Since DPOSEH is rare, only a few reports have described its risk factors. Anno et al. compared the clinical factors of 14 patients with early-onset and late-onset POSEH after posterior spinal surgery and found no significant difference between the two groups (16). Parthiban and colleagues reported a case of DPOSEH three days after T8 tumor resection. During the operation, a small paravertebral muscle artery rupture was found, which was considered to be related to the patient's overstretching of the paraspinal muscle (17). Another case of DPOSEH reported by Spanier et al. occurred on the 16th day after lumbar surgery. It was speculated that DPOSEH might be caused by heparin treatment of deep vein thrombosis of the right leg from the 12th day after the operation (18).

Most of the risk factors are focused on non-delayed SEH, among which Awad et al.'s study in 2005 was cited the most (19). Based on Awad et al.'s study and other literature, we summarized the risk factors into preoperative, intraoperative, and postoperative factors. Preoperative factors include age over 60 years (19, 20), RH positive blood group (19), hypertension (21), drinking (21), smoking (19), preoperative use of nonsteroidal anti-inflammatory drugs (NSAIDs) (19), preoperative coagulation dysfunction (history of hepatitis C and/or low platelet count (21), pregnancy (19, 21), previous spinal surgery (11), and minimally invasive procedures, such as epidural anesthesia (21). Intraoperative factors include multisegmental operation (22) and blood loss of >1,000 ml (19). Postoperative factors include postoperative INR of >2.0 within 48 h (19), postoperative hemoglobin level <10 g/dl within 48 h (19), postoperative ventilator dependence for >48 h (23), early postoperative intravenous heparin treatment (18), and tension-related events (tension, sneezing, constipation, sudden vigorous exercise, etc.) (15). However, some of these factors remain controversial. For example, preoperative use of NSAIDs and positive RH blood are common, and there is not enough evidence to support them as risk factors for POSEH (11). Awad et al. found that hypertension, drainage, and standard anticoagulation did not increase the risk of hematoma (19). Hirsh et al. suggested that the use of anticoagulants was better when started after 12 h (24). Another study showed that when low-molecular-weight heparins (LMWH) was used 24–36 h after spinal surgery, the risk of bleeding appeared to be low (25). The risk factors in our case included old age, RH-positive blood group, hypertension, smoking, low platelet count before the operation, and multi-stage operation. These factors might be the possible reasons for DPOSEH of this patient.

Moreover, other factors should also be considered. During the anesthesia resuscitation period, the intense activity of the neck may cause rebleeding of the coagulated blood vessels in the spinal canal. Therefore, the neck brace should be worn immediately after the operation, and surgeons should fully communicate with the anesthesiologist. Additionally, compression of the drainage tube is not suggested, which may induce a hematoma. In posterior spinal surgery, the supine position is not conducive to drainage (26).

## Rapid identification

### Clinical manifestations

POSEH after cervical spine surgery usually presents with severe neck pain and spasm, followed by progressive neurological deficits. Generally, lower limbs are heavier than upper limbs, and the tendon reflex is significantly weakened or disappears. Some patients have atypical manifestations, presenting only slight and slowly aggravating neurological deficits but with no pain, especially for DPOSEH (16, 19). A few cases have shown a gradual development of SEH after the operation, which may only manifest as neck pain and spasm without muscle strength reduction (14). Physical examination before the operation may indicate a suspected hematoma segment, which is helpful for rapid positioning and quick relief of compression during the operation.

### Imaging performance

Spinal epidural hematoma is diagnosed mainly using MRI. For SEH occurring within three days after the operation, MRI shows equal or slightly high signal in T1-weighted images and high or mixed signal in T2-weighted images. Biconvex or fusiform lesions can be found in sagittal or parasagittal reconstruction (27, 28). DPOSEH shows a high signal in T1- and T2-weighted images, which is mainly related to the deposition of hemosiderin. It was found that changes from equal to high signal on T1-weighted images are more valuable for DPOSEH diagnosis.

It is debatable whether MRI must be performed. Proponents believe that accurate imaging evidence is necessary before a second surgery. Owing to the onset time of DPOSEH, it is crucial to exclude other possible causes of spinal cord injury, such as vascular factors, infection, and internal fixation displacement (29). Additionally, psychological factors need to be evaluated. Yin et al. reported two cases of suspected POSEH with progressive paralysis after surgery. MRI confirmed no hematoma, but the patient was diagnosed with hysteria (30). The opponents believe that surgical exploration should be performed as early as the onset of neurological deficits, and MRI will certainly take a specific time and delay the operation time. Moreover, there are some hematomas in the epidural area after anterior cervical surgery, as discussed before, and the hemostatic materials used during surgery may also lead to false-positive results. We believe that MRI is very important for the differentiation of symptoms and diagnosis of DPOSEH. When we choose to perform MRI, we should fully consider that a possible delay in operation time may lead to a poor outcome of neurological function.

## Treatment methods

### Feasibility of conservative treatment

POSEH treatment includes conservative treatment and surgical treatment. For patients with severe neurological deficits, rapid deterioration of neurological function for a duration of <12 h, surgical treatment is still the first choice (31). However, conservative treatment can be attempted in cases with limited nerve damage and no progressive deterioration. Studies have shown that after conservative treatment, some patients' symptoms were relieved, the hematoma disappeared, and patients recovered completely (32, 33). However, such cases were mostly seen in a spontaneous extradural hematoma. Torres and colleagues used conservative treatment for an 80-year-old patient with C4-T1 SEH two hours after local block owing to his tendency to improve function. After one month, the hematoma was completely absorbed without residual neurological sequelae (34). Newey et al. reported a case of an 85-year-old patient with delayed SEH of C6-T1 three days after a traffic accident. After conservative treatment, the patient's symptoms improved three days later, and the patient completely recovered after three weeks (35).

### Methylprednisolone therapy

Methylprednisolone has been administered in patients with acute spinal cord injury. A high dose of methylprednisolone is beneficial to the occurrence of spinal cord impulse, increased blood flow, and reduction of lipid peroxidation and tissue degeneration of the spinal cord, resulting in a neuroprotective effect on acute spinal cord injury. It has also been used in SEH with a spinal cord injury (36). If neurological deficits improve with initial steroid treatment, we might consider not to perform surgery temporarily and observe closely instead. If surgery is needed, it could also be beneficial to administer methylprednisolone in the waiting and transitional periods. The administration of methylprednisolone was similar to that of a spinal cord injury. It was injected intravenously at 30 mg/kg within 15 min and maintained for 23 h at 5.4 mg/kg/h after 45-minute interval. Methylprednisolone therapy is generally considered to be effective within 72 h following the operation.

Ghaly et al. reported a POSEH case of incontinence 3 h after the local block of the cervical spine. Twenty minutes after methylprednisolone treatment, lower limbs and grip strength of the patient restored to grade 3, neck pain was significantly reduced, and rectal tension recovered (37). Cuenca et al. reported a case of spontaneous T4 SEH ten days after injury, and the patient was treated using high-dose intravenous methylprednisolone. Symptoms improved significantly (muscle strength from grade 0 to 2, perianal sensation, rectal tension, and penile abnormal erectile symptoms relieved) six minutes after the treatment. From the second day, the patient received dexamethasone 4 mg orally every 6 h and fully recovered after three days of treatment (38). Lin et al. reported two cases of traumatic cervical SEH with delayed quadriplegia treated with methylprednisolone. Dexamethasone (5 mg) was administered intravenously every 6 h. The muscle strength in one case decreased again 72 h after the effective use of dexamethasone; therefore, a C1-3 laminectomy was performed. The other case recovered seven days after dexamethasone therapy without requiring further surgical treatment (39).

### Timing of operation

The degree of neurological deficits before the operation and the timeliness of the operation are directly related to the outcome of SEH patients; therefore, the timing of the operation is quite important. Yi et al. analyzed nine cases of acute SEH and speculated a lighter neurological deficit or incomplete injury before the operation, and an earlier operation time resulted in a greater possibility of recovery (40). Lawton found that the recovery of neurological deficits in patients operated within 12 h was better than that after 12 h (7). Therefore, early detection and reoperation are beneficial for the recovery of neurological function.

### Key points of operation

Until now, the most used treatment option for POSEH was emergency surgery. The core steps included hematoma clearance, hemostasis, and drainage. First, implants, such as plates, screws, titanium meshes, or bone grafts, are removed, and then, the blood clot on the surface of the dura mater was removed. Thereafter, a thin ventricular drainage tube with side holes was placed at the back of the vertebral body near the decompression area to remove as many blood clots as possible. If there was blood seepage in the decompression bone surface, it should be carefully blocked again with bone wax. Bipolar electrocoagulation and gelatin sponge can be used in the cases of intraspinal venous plexus hemorrhage. In the case of active bleeding, double ligation should be performed on >1 mm thick blood vessels. Gelatin sponge, hemostatic gauze, or other hemostatic materials should not be placed between the dura mater and titanium mesh, if possible. When these items are used during the operation, it is recommended to remove them before incision closure. If the space between the titanium mesh or bone graft and the two sides of the vertebral body is small, it is difficult for the blood to flow from the epidural space to the drainage tube placed in the anterior space. It is recommended to bite off part of the bone to form a drainage channel. Finally, the original screws were replaced with renovation screws.

No significant correlation was found between POSEH and drainage tube placement. Patel et al. have reported that the drainage tube does not reduce the incidence rate of POSEH after spinal surgery (41). Furthermore, Kou et al. pointed out that drainage is not helpful in monitoring POSEH after posterior lumbar surgery (23). Additionally, Ahn et al. found that thicker drainage tubes could not prevent POSEH after posterior lumbar surgery than thin ones (42). However, these results may not be applicable to anterior cervical surgery. In our opinion, the drainage tube should be retained after the second removal of DPOSEH following anterior cervical surgery to reduce the accumulation of oozing blood.

In conclusion, DPOSEH could occur three days after anterior cervical surgery; therefore, the monitoring of neurological function is suggested to be extended. In addition to strengthening the knowledge of DPOSEH, doctors also need to standardize the diagnosis and treatment process to fully evaluate the preoperative, intraoperative, and postoperative risk factors and rapidly recognize and differentiate the changes in neurological symptoms (43). Methylprednisolone can be used reasonably during the transition period. If symptoms do not improve, emergency surgery should be performed as soon as possible. During the operation, the hematoma was removed, bleeding was stopped, and a drainage tube was inserted (44). After the operation, maintain stable anesthesia resuscitation and blood pressure, use a neck brace, and avoid tension and sudden exertion. If symptoms recur after the first evacuation operation, the operation is performed again if necessary.

## Data Availability

The original contributions presented in the study are included in the article/Supplementary Material, further inquiries can be directed to the corresponding author/s.
